# Association between labor epidural analgesia and gut microbiota: A prospective cohort study

**DOI:** 10.1016/j.heliyon.2024.e29883

**Published:** 2024-04-21

**Authors:** Jing-hui Hu, Jie Sheng, Hui-min Guo, Hong Liu, Xinyue Zhang, Bing Han, Ke Peng, Fu-hai Ji

**Affiliations:** a Departments of Anesthesiology, The First Affiliated Hospital of Soochow University, Suzhou, Jiangsu, China; b Institute of Anesthesiology, Soochow University, Suzhou, Jiangsu, China; c Department of Obstetrics and Gynecology, The First Affiliated Hospital of Soochow University, Suzhou, China; d Department of Anesthesiology and Pain Medicine, University of California Davis Health, Sacramento, CA, USA; e Suzhou Medical College of Soochow University, Suzhou, Jiangsu, China

**Keywords:** Labor epidural analgesia, Gut microbiota, Parturient, Neonate

## Abstract

**Background:**

Labor epidural analgesia (LEA) may influence gut microbiota. We explored the association between LEA and gut microbiota for both mothers and their newborns.

**Methods:**

In this prospective cohort study, parturients aged 25–35 years with a gestational age of 37–42 weeks and planned vaginal delivery were recruited. Twenty-one parturients received LEA (the LEA group), and 24 did not (the control group). Maternal and neonatal fecal samples were collected, and the gut microbiota profiles were analyzed using the 16S rRNA gene sequencing. The impact of LEA on gut microbiota was assessed using the general liner models.

**Results:**

We showcased the gut microbiota profile from the phyla to species levels based on data on 45 mother-newborn dyads. The results of α- and β-diversity suggested significant changes in gut microbiota between the LEA and control groups. After adjusting for baseline confounders, the administration of LEA had positive correlations with *R. ilealis* (β = 91.87, adjusted *P* = 0.007) in mothers; LEA also had negative correlations with *A. pittii* (β = −449.36, adjusted *P* = 0.015), *P. aeruginosa* (β = −192.55, adjusted *P* = 0.008), or *S. maltophilia* (β = −142.62, adjusted *P* = 0.001) in mothers, and with *Muribaculaceae* (β = −2702.77, adjusted *P* = 0.003) in neonates.

**Conclusion:**

LEA was associated with changes in maternal and neonatal gut microbiota, and future studies are still required to assess their impact on clinical outcomes and explore the mechanisms.

## 1 Introduction

As the largest microecosystem in human body, the gut is closely related to the health [1]. In recent years, the role of gut microbiota during the perinatal period has become a research spotlight. From the first trimester to the third trimester, the richness of maternal gut microbiota decreased and the diversity increased [2]. In the third trimester period, a study revealed that the top four most abundant phyla were *Bacillota*, *Bacteroidota*, *Pseudomonadota*, and *Actinomycetota*, and the abundant genera were *Prevotella*, *Faecalibacterium*, and unclassified *Lachnospiraceae* and *Ruminococcaceae* [3]. It was reported that the dysbiosis of gut microbiota could lead to adverse pregnancy outcomes, such as preterm birth, eclampsia, and postpartum hemorrhage [4,5]. For term neonates, the composition of gut microbiota mainly include the phyla *Pseudomonadota*, *Actinomycetota*, *Bacillota*, *Bacteroidota* and the genera *Escherichia*, *Bifidobacterium*, *Clostridium*, *Lactobacillus*, *Bacteroides*, *Prevotella* [6]. Gut microbiota plays a critical role in infant growth [7,8].

Many maternal exposures can affect maternal and neonatal gut microbiome [9]. Several studies showed that probiotic preparations led to continuous colonization of probiotics, while the differences in relative abundance disappear after cessation of supplementation [9]. Some studies found that emotions of pregnant women (e.g., stress, anxiety, and depression) reduced the diversity of neonatal gut microbiota, especially the decrease in the abundance of *s_dentium*, *s_longum*, and *s_Streptococcus_salivarius* [10]. Additionally, prenatal inflammatory led to changes in the abundance of *c_Clostridia* and *f_Ruminococcaceae* [11,12]. The use of antibiotic was associated with a relative reduction in the diversity of the neonatal microbiota [9]. Mitchell et al. found that delivery mode was a significant influencing factor for infant gut microbiota [13]. A proof-of-concept study showed the effects of fecal microbiota transplantation on microbial composition of infants delivered by cesarean section [14].

Labor epidural analgesia (LEA) is a widely-used technique for labor pain control. With the increase of LEA rate, its effects on mothers and babies have attracted people's attention. A meta-analysis showed that LEA can effectively relieve pain and improve maternal satisfaction, but it also had an increased risk of some complications such as assisted vaginal delivery and maternal fever [15]. However, some studies concluded that LEA did not increase the risk of mothers and neonates [16,17]. At present, LEA has a relatively positive effect, and the adverse events are mild and temporary [18].

LEA is also a kind of maternal exposure that can reduce stress for several hours. Recently, a study revealed that subarachnoid block with lidocaine could affect the gut microbiota to play a protective role in mice with colitis [19]. However, the impact of LEA on maternal-neonatal gut microbiota is unclear. Therefore, we designed this prospective cohort study to investigate the effects of LEA administration on maternal and neonatal gut microbiota.

## 2 Methods

### 2.1 Ethics and study design

This prospective cohort study was conducted at a tertiary hospital in Suzhou, China. The study protocol was approved by the Ethics Committee of the First Affiliated Hospital of Soochow University (Approval No. 2022–030). This study was conducted following the Declaration of Helsinki. All parturients provided their written informed consent.

### 2.2 Participants

To be included in this study, the following inclusion criteria should be met: (1) aged between 25 and 35 years; (2) natural conceived with a gestational age between 37 and 42 weeks; (3) singleton pregnancy with an expected birth weight of 2500–4000 g; (4) planned vaginal delivery; and (5) the habitual residence of Suzhou. The exclusion criteria included (1) using antibiotics or microecological preparations (e.g., probiotics, prebiotics, and synbiotics) within antepartum 3 months; (2) diabetes, immune disease, infectious disease, or digestive disease; (3) premature rupture of membranes; (4) history of anxiety or depressive disorders; or (5) declined to participate. The dropout criteria were as follows: (1) conversion to cesarean section, (2) withdrawal of consent, (3) intrapartum use of antibiotics, or (4) research stuff unavailable.

Upon parturients entered the delivery room for natural delivery, a researcher screened them for eligibility. Then, they signed the written informed consent and chose whether or not to receive LEA. The use of LEA was based on the parturients’ decision. Thus, parturients who did not receive LEA were included in the group C, while those who received LEA were included in the group E, with their newborns in the group C1 and group E1 respectively. Additionally, pudendal nerve block is routinely performed in labor analgesia at our institution.

### 2.3 Data collection

Participants’ clinical characteristics were collected from the electronic medical records and self-report questionnaires (**Supplemental file 1**), including demographics (i.e., age, gestational age, body mass index [BMI], parity, smoking status, diet over the past week, preoperative blood pressure, and prenatal stress and anxiety), labor data (i.e., highest temperature, use of oxytocin, duration of labor, intensity of pain, blood loss, and blood pressure), postpartum data (i.e., total blood loss, 2-h blood pressure, length of hospital stay, postpartum depression [PPD]), and neonatal data (i.e., fetal position, cord around neck, sex, weight, Apgar score, umbilical cord arterial blood pH, and Jaundice).

Diet (vegan diet, fermented vegetables, fried foods, and alcohol) over the past week, which was associated with gut microbiota [20,21] was collected via yes-or-no questions. Prenatal stress was evaluated using the 36-item Pregnancy Stress Rating Scale (PSRS36). The PRPS36 is a comprehensive self-report questionnaire with each item ranging from 0 to 4 (0 = none and 4 = very severe), and a higher score indicates a higher level of stress [22]. Anxiety was assessed using the Pregnancy Related Anxiety Questionnaire-Revised (PRAQ-R). The PRAQ-R is a 4-point Likert-type scale with 10 items (1 = not at all and 4 = very much in items 1–5; 1 = never and 4 = all of the time in items 6–10), and a higher score indicates a higher level of anxiety [23]. Intensity of pain was assessed using the numerical rating scale (NRS) (a total score of 0–10; 0 = no pain and 10 = the most severe pain). PPD was detected using the 10-item Edinburgh Postnatal Depression Scale (EPDS), with each item ranging from 0 to 3 and a cut-off score of 10 in the Chinese version [24]. To ensure the effectiveness, a research assistant gave explanations to help participants complete the questionnaires.

All parturients defecated immediately or shortly after delivery, and their fecal samples (∼5 g each) were collected. The neonatal first meconium samples (∼5 g each) were collected by the midwives within the first few hours of birth. The samples were stored using sterile containers with cryoprotectant in a −80 °C freezer before DNA extraction.

### 2.4 DNA extraction and amplification

Microbial DNA was extracted using the cetyltrimethylammonium bromide (CTAB) method. In brief, thawed samples were centrifuged, mixed with 1 mL CTAB and 20 μL lysozyme, and then completely lysed in a 65 °C water bath for 2 h. The supernatant (950 μL) was obtained after centrifugation, and mixed with the same volume of phenol (pH 8.0): chloroform: isoamyl alcohol (V25:24:1), followed by re-centrifugation at 12,000 rpm for 10 min. The chloroform: isoamyl alcohol (V24:1) was added into the supernatant and then re-centrifuged. The supernatant was mixed in isopropanol and precipitated at −20 °C. After centrifugation, the precipitates were washed twice with 1 mL 75 % ethanol. The dried DNA sample was dissolved with 51 μL ddH_2_O and treated with 1 μL RNase A for incubation at 37 °C for 15 min. The DNA concentration and purity was assessed using 1 % agarose gels. The DNA sample was diluted with sterile water to a final concentration of 1 ng/μL.

The 16S rRNA gene V4 region was amplified by the polymerase chain reaction (PCR) with primers 515-F (GTGCCAGCMGCCGCGGTAA) and 806-R (GGACTACHVGGGTWTCTAAT). The PCR reactions were carried out with 15 μL of Phusion® High-Fidelity PCR Master Mix (New England Biolabs), 2 μM of forward and reverse primers, and 10 ng template DNA. Thermal cycling consisted of an initial denaturation at 98 °C for 1 min, 30 cycles of denaturation at 98 °C for 10 s, annealing at 50 °C for 30 s, elongation at 72 °C for 30 s, and a final extension at 72 °C for 5 min. The PCR products were mixed with the same volume of 1X loading buffer (contained SYB green), followed by electrophoresis on 2 % agarose gel. The PCR mixture was purified with the Qiagen Gel Extraction Kit (Qiagen, Germany). During the PCR process, a negative control was used to control for contamination.

### 2.5 Sequencing processing

Sequencing libraries were generated using the TruSeq®DNA PCR-Free Sample Preparation Kit (Illumina, USA) following the manufacturer's instructions. After quality assessment, sequencing was performed on the Illumina NovaSeq platform, and 250 bp paired-end reads were generated. The barcode and primer sequences were removed, and the clean reads were obtained by the fastp (v0.22.0, https://github.com/OpenGene/fastp) and FLASH (v1.2.11, http://ccb.jhu.edu/software/FLASH/). The VSEARCH (v2.22.1, https://github.com/torognes/vsearch/) was used to detect and remove chimera sequences [25]. Finally, the effective tags were obtained. The filter level was operational taxonomic units (OTUs) with less than 2 tags in all samples [26].

### 2.6 OTU clusters and species annotation

The OTUs with ≥97 % similarity were clustered by the Uparse algorithm (USEARCH v7, http://drive5.com/uparse/) [27]. The representative sequence for each OTU was screened for species annotation in the Silva Database (http://www.arb-silva.de/) based on the Mothur algorithm [28]. The MAFFT (v7.520, https://mafft.cbrc.jp/alignment/software/) was used for multiple sequence alignment to construct phylogenetic relationships [29]. OTUs abundance information was normalized for a subsequent analysis by a standard of sequence number corresponding to the sample with the least sequences.

### 2.7 Statistical analysis

For the clinical characteristics, continuous variables were presented as mean ± SD or median (IQR) and analyzed using the *t*-test or Mann-Whitney *U* test according to the distribution, while categorical variables were expressed as number (%) and analyzed using the χ^2^ test or Fisher's exact test.

For the microbiota data, rationality of sequencing depth was tested by the rarefaction curve [30]. The rarefaction curve directly reflects the sample size of data sequenced and indirectly reflects the richness of species. When the curve tends to be flat, the amount of sequencing data is gradually reasonable, and more samples will only detect a few new species. The α-diversity indices (i.e., Chao 1 and Shannon) were analyzed by the Mann-Whitney *U* test. A higher value of Chao 1 represents a higher richness of the community, and a higher value of Shannon reflects a higher diversity of the community. The unweighted-unifrac distance values were converted and visualized by the principal coordinates analysis (PCoA) to show β-diversity, and the two groups were compared using the permutational multivariate analysis of variance (PERMANOVA) [31].

Then, the linear discriminant analysis (LDA) effect size (LEfSe) and Metastats were used to distinguish compositions within groups respectively. The LDA threshold was set to 4.0, with a larger score indicating a greater effect of statistical difference. The common differential bacteria of the two methods were used in further analyses. Based on the common differential bacteria, Spearman analysis was used to analyze the correlation between these bacteria and clinical factors (age, BMI, parity, PSRS36 scores, and PRAQ-R scores) [9,[32], [33], [34]] as well as intrapartum variables (highest temperature and duration of labor). Finally, a multivariable analysis of general liner model (GLM) was used to analyze the effect of LEA on gut microbiota with adjustment for possible confounders. The GLM β value indicates the changes in microbiota abundance by LEA.

A two-sided *P* < 0.05 indicates a statistical significance. Multiple comparisons in GLM were corrected using the Benjamini–Hochberg false discovery rate (FDR, with a threshold of adjusted *P* < 0.05). All data were analyzed using the R software (v4.2.2, www.R-project.org/) and the SPSS software (v26.0, IBM).

## 3 Results

### 3.1 Baseline and clinical characteristics

**Fig. 1:**
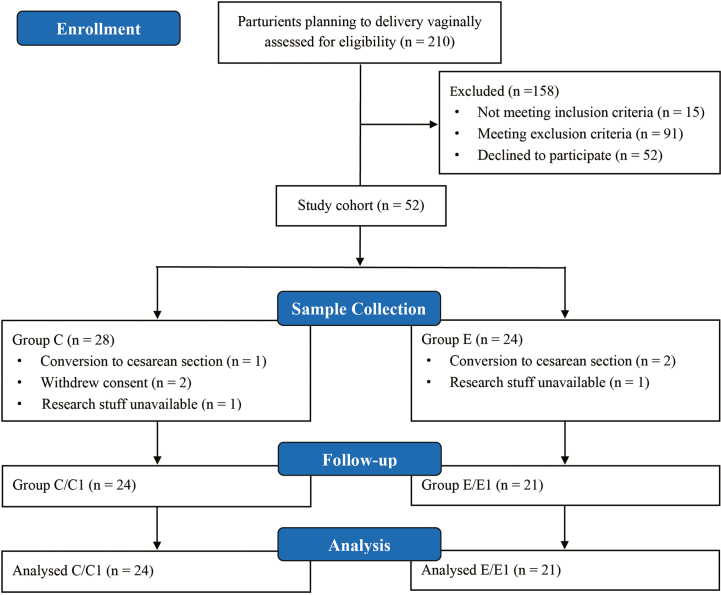
Study flow chart.

From March 7, 2022 to December 30, 2022, we initially screened 210 parturients (Fig. 1). Of them, 158 parturients were excluded, and 52 were enrolled in this study. Subsequently, 7 parturients were excluded due to conversion to cesarean section (n = 3), withdrew consent (n = 2), and research stuff unavailable (n = 2). No parturients were excluded due to intrapartum use of antibiotics. Finally, 45 pairs of parturients and neonates were included and analyzed (n = 24 in the group C/C1 and n = 21 in the group E/E1).

The maternal characteristics are shown in Table 1. The age of parturients was 29.8 ± 3.3 years in the group C vs. 27.6 ± 2.8 years in the group E (*P* = 0.018). The gestational age was 279 (273.5, 284) and 278 (273.5, 282) days in the group C and group E, respectively. Eight of 24 (33.3 %) parturients were nulliparas in the group C, while 17 of 21 (81 %) were nulliparas in the group E (*P* = 0.001). The participants in the group E had higher PSRS36 scores than those in the group C (*P* = 0.007), while the two groups had similar PRAQ-R scores. The group E had a longer duration of labor in stage 1 (*P* = 0.010) and stage 2 (*P* = 0.005). The highest NRS pain scores were significantly lower in the group E compared to the group C (median [IQR], 2 [2, 3] vs. 9.5 [8, 10]; *P* < 0.001). One patient in the group C and 2 patients in the group E showed PPD at 6 postpartum weeks, and no patients had PPD at 6 months.

**Table 1:** Maternal and neonatal characteristics.

Variables	Group C/C1 (n = 24)	Group E/E1 (n = 21)	*P* value
*Maternal baseline*			
**Age, y**	29.8 ± 3.3	27.6 ± 2.8	0.018
**BMI, kg/m**^**2**^	26.3 ± 2.9	27.6 ± 2.6	0.102
**Gestational age, d**	279 (273.5, 284)	278 (273.5, 282)	0.546
**Parity**			
**Nulliparas**	8 (33.3 %)	17 (81 %)	0.001
**Multiparas**	16 (66.7 %)	4 (19 %)	
**History of smoking**	0	0	1.000
**Diet over the past week**			
**Vegan diet**	0	0	1.000
**Fermented vegetables**	12 (50 %)	6 (28.6 %)	0.143
**Fried foods**	2 (8.3 %)	1 (4.8 %)	1.000
**Alcohol**	4 (16.7 %)	1 (4.8 %)	0.428
**Preoperative SBP, mmHg**	115.13 ± 10.69	119.19 ± 11.06	0.217
**Preoperative DBP, mmHg**	72.29 ± 7.50	74.1 ± 5.81	0.378
**PSRS36 scores**	11 (0, 22.25)	23 (10.5, 41)	0.007
**PRAQ-R scores**	15 (10, 20)	16 (12.5, 22.5)	0.186
*Intrapartum*			
**Use of Oxytocin**	11 (45.8 %)	14 (66.7 %)	0.161
**Highest temperature, °C**	37 (36.9, 37.2)	37.2 (37.1, 37.3)	0.001
**Duration of labor, min**			
**Stage 1**	305 (232.5, 360)	420 (292.5, 540)	0.010
**Stage 2**	23.5 (10, 45.8)	50 (30, 88)	0.005
**Stage 3**	5 (3.3, 7.8)	5 (4, 7)	0.662
**Highest NRS pain scores**	9.5 (8, 10)	2 (2, 3)	<0.001
**Blood loss, ml**	155 (120, 237.5)	200 (100, 225)	0.737
**SBP, mmHg**	120 (112.25, 122.75)	113 (111.5, 121.5)	0.304
**DBP, mmHg**	70 (65, 75)	69 (64, 73.5)	0.359
*Postpartum*			
**Total blood loss, ml**	200 (167.5, 227.5)	240 (140, 285)	0.837
**2-h SBP, mmHg**	116 ± 7.05	117.1 ± 7.96	0.627
**2-h DBP, mmHg**	70.21 ± 5.07	68.95 ± 6.56	0.474
**Length of hospital stay**	3 (3, 4)	3 (3, 4)	0.563
**6-week EPDS scores**	2 (0, 5)	4 (0.5, 5.5)	0.283
**6-week PPD**^a^	1 (4.2 %)	2 (9.5 %)	0.472
**6-month EPDS scores**	0 (0, 1.75)	0 (0, 1)	0.968
**6-month PPD**^a^	0	0	1.000
*Neonatal characteristics*			
**Fetal position, LOA**	24 (100 %)	21 (100 %)	1.000
**Cord around neck**	5 (20.8 %)	6 (28.6 %)	0.547
**Sex**			
**Male**	13 (54.2 %)	13 (61.9 %)	0.600
**Female**	11 (45.8 %)	8 (38.1 %)	
**Birth weight, g**	3427.1 ± 346.4	3311.9 ± 298.7	0.242
**1-min Apgar scores**	9.95 ± 0.22	10	0.329
**5-min Apgar scores**	10	10	1.000
**Umbilical cord arterial blood** pH	7.27 ± 0.11	7.25 ± 0.08	0.464
**Jaundice**	4 (16.7 %)	8 (38.1 %)	0.105

Data are mean ± SD, median (IQR), n (%).

BMI, body mass index; SBP, systolic blood pressure; DBP, diastolic blood pressure; PSRS36, 36-item pregnancy stress rating scale; PRAQ-R, pregnancy related anxiety questionnaire-revised; NRS, numerical rating scale; EDPS, Edinburgh postnatal depression scale; PPD, postpartum depression; LOA, left occiput anterior.

a Defined as EPDS score ≥10.

The two groups were comparable in terms of the neonatal characteristics (Table 1). Only one newborn baby had 1-min Apgar score of 9. All neonates had an Apgar score of 10 at 5 min. The umbilical cord arterial blood pH value was 7.27 ± 0.11 and 7.25 ± 0.08 in the group C1 and group E1, respectively. Four newborns (16.7 %) in the group C1 and 8 (38.1 %) in the group E1 developed neonatal jaundice, without a significant between-group difference.

### 3.2 Gut microbial diversity

**Fig. S1:**
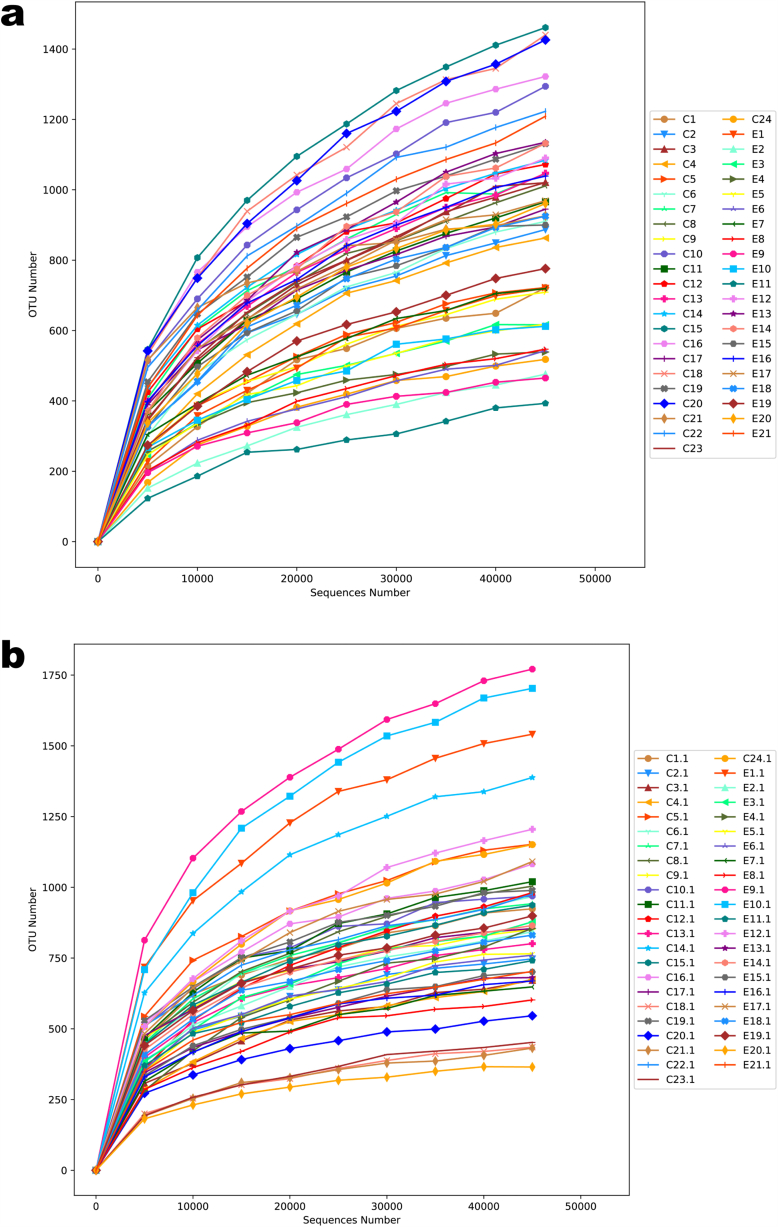
Rarefaction curve. (**a**) maternal gut microbiota composition; (**b**) neonatal gut microbiota composition.

**Fig. S2:**
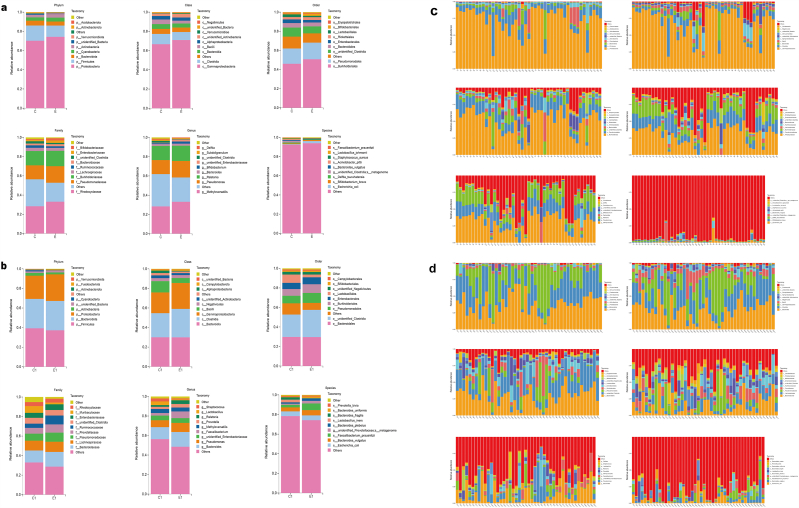
Stacked bar charts of the top 10 dominant bacteria from the phylum to species levels: (**a**) maternal gut microbiota; (**b**) neonatal gut microbiota; (**c**) maternal gut microbiota composition among samples; (**d**) neonatal gut microbiota composition among samples. C, control (maternal); E; epidural analgesia (maternal); C1, control (neonatal); E1; epidural analgesia (neonatal).

After data trimming and quality filtering, 4719 OTUs in the maternal samples and 4793 in the neonatal samples (**Supplemental data 1**) were delineated. The sequencing depth was reasonable according to the rarefaction curve (Fig. S1, Supplemental data 2). The stacked bar charts showed the top 10 dominant bacteria from the phylum level to species level of the two groups and among individual samples (Fig. S2).

**Fig. 2:**
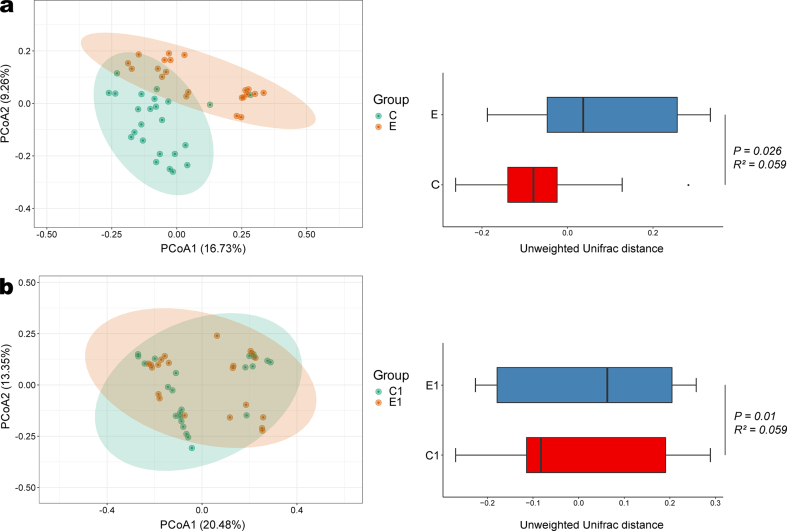
Visualization of β-diversity by PCoA. (**a**) Maternal gut microbiota. (**b**) Neonatal gut microbiota. The ellipses represent the 95 % confidence intervals, and the boxplot represents the distribution of two groups of samples on the PCoA1 axis. PCoA, principal coordinates analysis; C, control (maternal); E; epidural analgesia (maternal); C1, control (neonatal); E1; epidural analgesia (neonatal).

The values of α-diversity indices, including Chao 1 and Shannon, are shown in Table 2. The parturients in the group C had significantly higher values of Chao 1 (*P* = 0.007) and Shannon (*P* = 0.026) compared with the group E, while the neonates of the two groups had similar α-diversity values. Fig. 2 shows the β-diversity based on the converted unweighted-unifrac distance values (the initial values available in **supplemental data 3**). Furthermore, according to the results of PERMANOVA, there was a significant shift in both mothers (*P* = 0.026, R^2^ = 0.059; Fig. 2a) and neonates (*P* = 0.01, R^2^ = 0.059; Fig. 2b).

**Table 2:** α-diversity values of two groups.

Variables	Group C (n = 24)	Group E (n = 21)	*P* value
*Maternal*			
**Chao1**	1364.10 (1180.01, 1614.04)	989.05 (775.36, 1399.48)	0.007
**Shannon**	4.07 (3.65, 5.65)	3.79 (2.88, 4.37)	0.026
*Neonatal*			
**Chao1**	1197.31 (930.87, 1329.58)	1039.72 (861.40, 1319.21)	0.539
**Shannon**	6.45 (5.08, 6.87)	5.98 (5.14, 6.53)	0.387

### 3.3 Differential bacteria analysis

The results of LEfSe distinguished taxa from the levels of phylum to species between the groups (Table S1). Considering that LEfSe is testing at multiple levels and a difference at one level can bleed to others, we applied Metastats for testing at different levels to reduce this effect (Tables S2 and S3). There are 9 common differential bacteria between the LEfSe and Metastats results. Among those, *g_unidentified_Clostridia;s_metagenome* was excluded from further analysis due to unidentification. The maternal results showed that species *Acinetobacter pittii*, *Pseudomonas aeruginosa*, and *Stenotrophomonas maltophilia* were abundant in the group C, while the species *Romboutsia ilealis* were abundant in the group E. The neonatal results revealed that classes *Bacilli* and families *Muribaculaceae* and *Lactobacillaceae*, genus *Lactobacillus* were dominant in the group C1.

### 3.4 Association between LEA and gut microbiota

**Fig. 3:**
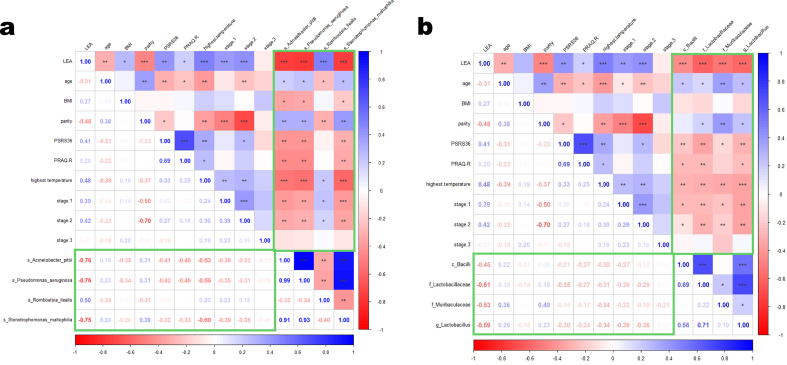
The correlations between clinical characteristics and differential bacteria. (**a**) Maternal gut microbiota. (**b**) Neonatal gut microbiota. Blue color represents a positive correlation, red color represents a negative correlation, and darkness is in line with the degree of corrlation. Numbers are the values of correlation coefficients. Green boxes highlight the coefficients and statistical significance for the corrlations. LEA, labor epidural analgesia; BMI, body mass index. *P < 0.05, **P < 0.05, ***P < 0.001. (For interpretation of the references to color in this figure legend, the reader is referred to the Web version of this article.)

The correlations between different exposure factors (e.g., LEA, age, BMI, parity, PSRS36 scores, PRAQ-R scores, highest temperature and duration of labor) and differential bacteria are shown in Fig. 3. In mothers (Fig. 3a), *A. pittii*, *P. aeruginosa* and *S. maltophilia* were positively correlated with age and parity (*P* < 0.05), and negatively correlated with the other factors (*P* < 0.05) except for labor stage 3; *R. ilealis* was positively correlated with LEA, highest temperature, labor stage 1 and stage 2 (*P* < 0.05), and negatively correlated with age and parity (*P* < 0.05). In neonates (Fig. 3b), all four bacteria (*Bacilli*, *Lactobacillaceae*, *Muribaculaceae* and *Lactobacillus*) had negative correlations with LEA, PSRS36 scores, PRAQ-R scores, highest temperature, stage 1 and stage 2 (*P* < 0.05), and a positive correlation with age (*P* < 0.05); moreover, *Lactobacillaceae*, *Muribaculaceae* and *Lactobacillus* had positive correlations with parity (*P* < 0.05).

Table 3 shows the results of GLM adjusting for the above-mentioned confounders. The maternal results showed that LEA had a significantly positive correlation with *R. ilealis* (β = 91.87, *P* = 0.003, adjusted *P* = 0.007), and negative correlations with *A. pittii* (β = −449.36, *P* = 0.015, adjusted *P* = 0.015), *P. aeruginosa* (β = −192.55, *P* = 0.006, adjusted *P* = 0.008), and *S. maltophilia* (β = −142.62, *P* < 0.001, adjusted *P* = 0.001). Among those 4 bacteria, *R. ilealis* has been shown to be associated with a nutrient-rich environment [35], while the other three are opportunistic pathogens [[36], [37], [38]]. The neonatal results suggested negative correlations were found between LEA and *f_Muribaculaceae* (β = −2702.77, *P* = 0.001, adjusted *P* = 0.003), which is considered to have anti-inflammatory effects [39].

**Table 3:** Relation between LEA and gut microbiota using GLM.

Taxonomy	LEA		
	Β *value*	*P value*	*Adjusted P value*
*Maternal*			
***s__Acinetobacter_pittii***	−449.36	0.015	0.015
***s__Pseudomonas_aeruginosa***	−192.55	0.006	0.008
***s__Romboutsia_ilealis***	91.87	0.003	0.007
***s__Stenotrophomonas_maltophilia***	−142.62	<0.001	0.001
*Neonatal*			
***c__Bacilli***	−2928.31	0.067	0.134
***f__Lactobacillaceae***	−1505.12	0.110	0.146
***f__Muribaculaceae***	−2702.77	0.001	0.003
***g__Lactobacillus***	−908.16	0.341	0.341

LEA, labour epidural analgesia; GLM, multivariable analysis of general liner model; *p*, phylum; *c*, class; *o*, order; *f*, family; *g*, genus; *s*, species.

## 4 Discussion

Our results showcased the composition of gut microbiota at different levels for both mothers and neonates. Compared with the control group, the parturients receiving LEA had a lower α-diversity values, a significantly higher abundance of *R. ilealis* and lower abundances of *A. pittii*, *P. aeruginosa* and *S. maltophilia* at the species level. The neonates in the LEA group had a lower abundance of the family *Muribaculaceae*.

The α-diversity results indicated that the diversity and richness of gut microbiota in the group E were significantly lower than those in the group C. Nevertheless, the clinical implications of these differences may be difficult to interpret. A previous observational study showed that gestational diabetes, pre-obesity, and obesity were associated with lower α-diversity values [40], while Crusell et al. did not find such an association [41]. In addition, the changes in α-diversity with the progress of pregnancy were not consistent in recent studies [2,42,43]. In our study, although the richness and diversity of maternal microbiota decreased in parturients receiving LEA, the clinical outcomes were comparable between the two groups. The β-diversity analysis showed the between-group differences in compositions of gut microbiota, and then the differential analysis was conducted to detect the specific bacteria associated with LEA.

According to the changes in maternal gut microbiota (increased *R. ilealis* and reduced *A. pittii*, *P. aeruginosa* and *S. maltophilia*), the parturients may benefit from LEA. *R. ilealis* was an obligately anaerobic bacterium and firstly isolated from the rat digestive system in 2014 [44]. Researchers revealed that *R. ilealis* CRIB^T^ utilized carbohydrates through different and partially redundant pathways to adapt to the small intestine [35]. Rodrigues et al. performed a study on both type-2-diabetes-like mice and humans to show that *R. ilealis* was a possible pathogen associated with worsened glucose metabolism [45]. At present, however, studies on *R. ilealis* are still limited. The other three species *A. pittii*, *P. aeruginosa* and *S. maltophilia* are opportunistic pathogens, which could lead to antibiotic-tolerant bacterial infections [[36], [37], [38]]. A meta-analysis suggested that the estimated rate of maternal peripartum infection was almost 4 % [46]. Therefore, LEA might have a potential protective effect on parturients.

The changes in neonatal gut microbiota (reduced *Muribaculaceae*) suggested that the newborns could be affected by LEA. *Muribaculaceae*, used to be called S24–7, has a functional diversity in the complex carbohydrates degradation [47]. Volk et al. found that the barrier function of the inner mucus layer depended on *Muribaculacea* to some extent in mouse model [48]. De et al. found that Muribaculacea was enriched in mice with colitis and involved in repair [49]. An observational study showed a significant enrichment of *Muribaculaceae* in infants with respiratory syncytial virus [50]. Up to date, the research on *Muribaculaceae* is still in progress. In our study, the effect of LEA on neonatal gut microbiota was not clear, and there were no adverse clinical outcomes.

The brain-gut-microbiome axis has been confirmed by a vast of preclinical evidence, which reveals the interaction of the central nervous system and gut microbiome [[51], [52], [53]]. Stress can affect the intestinal homeostasis by this axis, including endocrine (the hypothalamic-pituitary-adrenal axis) and neural pathways (the sympathetic and parasympathetic nervous systems and enteric nervous system) [[54], [55], [56]]. The outcomes depend on the frequency, duration and intensity of exposure [57]. Researchers observed that a stress condition for 4–6 h led to the reduction of immunoglobulin A levels and the increase of inflammations in intestinal injury models [58,59]. Rodent and human studies implicated that maternal stressor exposure could affect the offspring gut microbiome [60]. Moreover, Yu et al. found that subarachnoid block with lidocaine was able to alleviate clinical symptoms in mice with colitis by regulating the gut microbiota [19]. Therefore, we inferred that labor pain led to persistent acute stress, and LEA can block the afferent stimulus and the efferent sympathetic nerve to reduce the stress response which ultimately affects maternal and neonatal gut microbiota. In addition, Loftus et al. found that a certain concentration of fentanyl or sufentanil could be measured in the maternal vein, umbilical artery and umbilical vein [61], so the delivery of anesthetics through the placental barrier to the fetus may also affect the gut microbiota.

In addition, studies have shown the association between microbiota and pregnancy outcomes (such as intrapartum pyrexia, chorioamnionitis, and preterm labor). For instance, certain microorganisms like Fusobacterium species, Streptococcus thermophilus, and Bergeyella have been identified as potential causes of preterm labor and chorioamnionitis [62]. Notably, oral microbes such as Streptococcus agalactiae and *Escherichia coli* detected on placenta through 16S sequencing, and vaginal microbes like Lactobacillus jensenii, Prevotella bivia, Prevotella spp., A. vaginae, F. magna, and Aerococcus christensenii are associated with these complications [63,64].

As the first study on the association between LEA and gut microbiota, our study provides a novel insight into the effects of LEA on maternal and neonatal outcomes However, there are several limitations. First, we did not collect detailed data on maternal diet which may influence the baseline gut microbiota. Second, we did not collect prenatal samples of gut microbiota in the parturients, so we cannot compare these baseline microbiota data with that after delivery. Third, there is no indication that the changes seen in this study actually persist beyond day 0 in neonates as only the meconium microbiota was assessed. Fourth, the study of gut microbiota typically requires a minimum of 30 samples per group. Although the rarefaction curve suggests that current sample size seemed to be adequate, this is a single-center study with a small sample size. Fifth, there should be considerations for opioid consumption or an effect dose response in the future, such as increased microbiota changes with increasing use of LEA. Sixth, the OTU clustering is a typical method that has been widely used in many studies, so we used OUT method in our study. However, utilizing the amplicon sequence variant method would likely yield more accurate results. Finally, compared with absolute quantification, the relative abundance analysis based on 16S rRNA sequences was less sensitive to show the differences in gut microbiota.

In conclusion, LEA was associated with changes in maternal and neonatal gut microbiota. For parturients receiving LEA, the abundance of opportunistic pathogens decreased, and *R. ilealis* abundance increased. The abundance of *Muribaculaceae* decreased in neonates whose mothers receiving LEA. In the future, it is necessary to verify our findings in a larger cohort and explore the mechanisms using multi-omics metabolism analysis.

## Ethics and dissemination

This study was approved by the Ethics Committee of the First Affiliated Hospital of Soochow University (Approval No. 2022–030). All parturients provided their written informed consent.

## Funding

This work was supported by the Suzhou Medical Health Science and Technology Innovation Project (SKY2022136 to KP), Jiangsu Medical Association Anesthesia Research Project (SYH-32021-0036 (2021031) to KP), 10.13039/501100001809National Natural Science Foundation of China (82072130 to FHJ), Key Medical Research Projects in 10.13039/501100002949Jiangsu Province (ZD2022021 to FHJ), and Suzhou Clinical Medical Center for Anesthesiology (Szlcyxzxj202102 to FHJ). The funding organizations had no role in the design and conduct of the study; collection, management, analysis, and interpretation of the data; preparation, review, or approval of the manuscript; and decision to submit the manuscript for publication.

## Data availability statement

The raw sequence data reported in this paper have been deposited in the Genome Sequence Archive (Genomics, Proteomics & Bioinformatics 2021) in National Genomics Data Center (Nucleic Acids Res 2022), China National Center for Bioinformation/Beijing Institute of Genomics, Chinese Academy of Sciences (GSA: CRA013044) that are publicly accessible at https://ngdc.cncb.ac.cn/gsa.

## CRediT authorship contribution statement

**Jing-hui Hu:** Writing – original draft, Investigation, Formal analysis, Data curation. **Jie Sheng:** Writing – original draft, Formal analysis, Data curation. **Hui-min Guo:** Supervision, Data curation. **Hong Liu:** Supervision, Data curation, Conceptualization. **Xinyue Zhang:** Formal analysis, Data curation. **Bing Han:** Supervision, Data curation. **Ke Peng:** Writing – review & editing, Validation, Supervision, Project administration, Conceptualization. **Fu-hai Ji:** Writing – review & editing, Supervision, Conceptualization.

## Declaration of competing interest

The authors declare that they have no known competing financial interests or personal relationships that could have appeared to influence the work reported in this paper.

## References

[1] Fan Y., Pedersen O. (2021). Gut microbiota in human metabolic health and disease. Nat. Rev. Microbiol..

[2] Koren O., Goodrich J.K., Cullender T.C. (2012). Host remodeling of the gut microbiome and metabolic changes during pregnancy. Cell.

[3] Tang M., Weaver N.E., Frank D.N. (2022). Longitudinal reduction in diversity of maternal gut microbiota during pregnancy is observed in multiple Low-Resource settings: results from the women first trial. Front. Microbiol..

[4] Li C., Liu C., Li N. (2022). Causal associations between gut microbiota and adverse pregnancy outcomes: a two-sample Mendelian randomization study. Front. Microbiol..

[5] Qi X., Yun C., Pang Y., Qiao J. (2021). The impact of the gut microbiota on the reproductive and metabolic endocrine system. Gut Microb..

[6] Princisval L., Rebelo F., Williams B.L. (2021). Association between the mode of delivery and infant gut microbiota composition up to 6 Months of age: a systematic literature review considering the role of breastfeeding. Nutr. Rev..

[7] Nguyen T.T.B., Chung H.J., Kim H.J., Hong S.T. (2019). Establishment of an ideal gut microbiota to boost healthy growth of neonates. Crit. Rev. Microbiol..

[8] Ihekweazu F.D., Versalovic J. (2018). Development of the pediatric gut microbiome: impact on health and disease. Am. J. Med. Sci..

[9] Grech A., Collins C.E., Holmes A. (2021). Maternal exposures and the infant gut microbiome: a systematic review with meta-analysis. Gut Microb..

[10] Galley J.D., Mashburn-Warren L., Blalock L.C. (2023). Maternal anxiety, depression and stress affects offspring gut microbiome diversity and bifidobacterial abundances. Brain Behav. Immun..

[11] Juckel G., Manitz M.P., Freund N., Gatermann S. (2021). Impact of Poly I:C induced maternal immune activation on offspring's gut microbiome diversity - implications for schizophrenia. Prog. Neuro-Psychopharmacol. Biol. Psychiatry.

[12] Hsiao E.Y., McBride S.W., Hsien S. (2013). Microbiota modulate behavioral and physiological abnormalities associated with neurodevelopmental disorders. Cell.

[13] Mitchell C.M., Mazzoni C., Hogstrom L. (2020). Delivery mode affects stability of early infant gut microbiota. Cell Rep Med.

[14] Korpela K., Helve O., Kolho K.L. (2020). Maternal fecal microbiota transplantation in cesarean-born infants rapidly restores normal gut microbial development: a proof-of-concept study. Cell.

[15] Anim-Somuah M., Smyth R.M., Cyna A.M., Cuthbert A. (2018). Epidural versus non-epidural or no analgesia for pain management in labour. Cochrane Database Syst. Rev..

[16] 谢星 张丽芳 (2018). 椎管内分娩镇痛对足月初产妇产程进展及分娩结局的影响. 现代妇产科进展.

[17] Liu X., Zeng R., Chen Q., Ke D., Zhu Z. (2021).

[18] Callahan E.C., Lee W., Aleshi P., George R.B. (2023). Modern labor epidural analgesia: implications for labor outcomes and maternal-fetal health. Am. J. Obstet. Gynecol..

[19] Hong Y., Zhao J., Chen Y.R. (2022). Spinal anesthesia alleviates dextran sodium sulfate-induced colitis by modulating the gut microbiota. World J. Gastroenterol..

[20] Galena A.E., Chai J., Zhang J. (2022). The effects of fermented vegetable consumption on the composition of the intestinal microbiota and levels of inflammatory markers in women: a pilot and feasibility study. PLoS One.

[21] Wang Y., Xie T., Wu Y., Liu Y., Zou Z., Bai J. (2021). Impacts of maternal diet and alcohol consumption during pregnancy on maternal and infant gut microbiota. Biomolecules.

[22] Chen C.H. (2015). Revision and validation of a scale to assess pregnancy stress. J. Nurs. Res..

[23] Chan C.Y., Lee A.M., Koh Y.W., Tang C.S.K. (2020). Validation of the Chinese version of the Pregnancy-related Anxiety Questionnaire-Revised (PRAQ-R) and its distinction from general anxiety and depression in pregnant women. J. Psychosom. Obstet. Gynaecol..

[24] Lee D.T., Yip S.K., Chiu H.F. (1998). Detecting postnatal depression in Chinese women. Validation of the Chinese version of the Edinburgh Postnatal Depression Scale. Br. J. Psychiatry.

[25] Rognes T., Flouri T., Nichols B., Quince C., Mahe F. (2016). VSEARCH: a versatile open source tool for metagenomics. PeerJ.

[26] Bokulich N.A., Subramanian S., Faith J.J. (2013). Quality-filtering vastly improves diversity estimates from Illumina amplicon sequencing. Nat. Methods.

[27] Edgar R.C. (2013). UPARSE: highly accurate OTU sequences from microbial amplicon reads. Nat. Methods.

[28] Schloss P.D., Westcott S.L., Ryabin T. (2009). Introducing mothur: open-source, platform-independent, community-supported software for describing and comparing microbial communities. Appl. Environ. Microbiol..

[29] Katoh K., Misawa K., Kuma K., Miyata T. (2002). MAFFT: a novel method for rapid multiple sequence alignment based on fast Fourier transform. Nucleic Acids Res..

[30] Gotelli N.J., Colwell R.K. (2001). Quantifying biodiversity: procedures and pitfalls in the measurement and comparison of species richness. Ecol. Lett..

[31] Anderson M.J. (2008). A new method for non‐parametric multivariate analysis of variance. Austral Ecol..

[32] Kennedy K.M., Plagemann A., Sommer J. (2023). Parity modulates impact of BMI and gestational weight gain on gut microbiota in human pregnancy. Gut Microb..

[33] Berry A.S.F., Pierdon M.K., Misic A.M. (2021). Remodeling of the maternal gut microbiome during pregnancy is shaped by parity. Microbiome.

[34] Zijlmans M., Korpela K., Riksen-Walraven J., de Vos W., de Weerth C. (2015). Maternal prenatal stress is associated with the infant intestinal microbiota. Psychoneuroendocrinology.

[35] Gerritsen J., Hornung B., Renckens B. (2017). Genomic and functional analysis of Romboutsia ilealis CRIB(T) reveals adaptation to the small intestine. PeerJ.

[36] Brooke J.S. (2012). Stenotrophomonas maltophilia: an emerging global opportunistic pathogen. Clin. Microbiol. Rev..

[37] Wisplinghoff H., Paulus T., Lugenheim M. (2012). Nosocomial bloodstream infections due to Acinetobacter baumannii, Acinetobacter pittii and Acinetobacter nosocomialis in the United States. J. Infect..

[38] Qin S., Xiao W., Zhou C. (2022). Pseudomonas aeruginosa: pathogenesis, virulence factors, antibiotic resistance, interaction with host, technology advances and emerging therapeutics. Signal Transduct. Targeted Ther..

[39] Shang L., Liu H., Yu H. (2021). Core altered microorganisms in colitis mouse model: a comprehensive time-point and fecal microbiota transplantation analysis. Antibiotics (Basel).

[40] Abdullah B., Daud S., Aazmi M.S., Idorus M.Y., Mahamooth M.I.J. (2022). Gut microbiota in pregnant Malaysian women: a comparison between trimesters, body mass index and gestational diabetes status. BMC Pregnancy Childbirth.

[41] Crusell M.K.W., Hansen T.H., Nielsen T. (2018). Gestational diabetes is associated with change in the gut microbiota composition in third trimester of pregnancy and postpartum. Microbiome.

[42] Li M., Zhang G., Cui L. (2023). Dynamic changes in gut microbiota during pregnancy among Chinese women and influencing factors: a prospective cohort study. Front. Microbiol..

[43] Ferrocino I., Ponzo V., Gambino R. (2018). Changes in the gut microbiota composition during pregnancy in patients with gestational diabetes mellitus (GDM). Sci. Rep..

[44] Gerritsen J., Fuentes S., Grievink W. (2014). Characterization of Romboutsia ilealis gen. nov., sp. nov., isolated from the gastro-intestinal tract of a rat, and proposal for the reclassification of five closely related members of the genus Clostridium into the genera Romboutsia gen. nov., Intestinibacter gen. nov., Terrisporobacter gen. nov. and Asaccharospora gen. nov. Int. J. Syst. Evol. Microbiol..

[45] Rodrigues R.R., Gurung M., Li Z. (2021). Transkingdom interactions between Lactobacilli and hepatic mitochondria attenuate western diet-induced diabetes. Nat. Commun..

[46] Woodd S.L., Montoya A., Barreix M. (2019). Incidence of maternal peripartum infection: a systematic review and meta-analysis. PLoS Med..

[47] Lagkouvardos I., Lesker T.R., Hitch T.C.A. (2019). Sequence and cultivation study of Muribaculaceae reveals novel species, host preference, and functional potential of this yet undescribed family. Microbiome.

[48] Volk J.K., Nystrom E.E.L., van der Post S. (2019). The Nlrp6 inflammasome is not required for baseline colonic inner mucus layer formation or function. J. Exp. Med..

[49] De A., Chen W., Li H. (2021). Bacterial swarmers enriched during intestinal stress ameliorate damage. Gastroenterology.

[50] Harding J.N., Siefker D., Vu L. (2020). Altered gut microbiota in infants is associated with respiratory syncytial virus disease severity. BMC Microbiol..

[51] Osadchiy V., Martin C.R., Mayer E.A. (2019). Gut microbiome and modulation of CNS function. Compr. Physiol..

[52] Gershon M.D., Margolis K.G. (2021). The gut, its microbiome, and the brain: connections and communications. J. Clin. Invest..

[53] Fung T.C., Olson C.A., Hsiao E.Y. (2017). Interactions between the microbiota, immune and nervous systems in health and disease. Nat. Neurosci..

[54] Brzozowski B., Mazur-Bialy A., Pajdo R. (2016). Mechanisms by which stress affects the experimental and clinical inflammatory bowel disease (IBD): role of brain-gut Axis. Curr. Neuropharmacol..

[55] Guzman-Mejia F., Godinez-Victoria M., Vega-Bautista A., Pacheco-Yepez J., Drago-Serrano M.E. (2021). Intestinal homeostasis under stress siege. Int. J. Mol. Sci..

[56] Yoo B.B., Mazmanian S.K. (2017). The enteric network: interactions between the immune and nervous systems of the gut. Immunity.

[57] Buynitsky T., Mostofsky D.I. (2009). Restraint stress in biobehavioral research: recent developments. Neurosci. Biobehav. Rev..

[58] Gong Y., Niu W., Tang Y. (2019). Aggravated mucosal and immune damage in a mouse model of ulcerative colitis with stress. Exp. Ther. Med..

[59] Ponferrada A., Caso J.R., Alou L. (2007). The role of PPARgamma on restoration of colonic homeostasis after experimental stress-induced inflammation and dysfunction. Gastroenterology.

[60] Yeramilli V., Cheddadi R., Shah J., Brawner K., Martin C. (2023). A review of the impact of maternal prenatal stress on offspring microbiota and metabolites. Metabolites.

[61] Loftus J.R., Hill H., Cohen S.E. (1995). Placental transfer and neonatal effects of epidural sufentanil and fentanyl administered with bupivacaine during labor. Anesthesiology.

[62] Fox C., Eichelberger K. (2015). Maternal microbiome and pregnancy outcomes. Fertil. Steril..

[63] Zhao F., Hu X., Ying C. (2023). Advances in research on the relationship between vaginal microbiota and adverse pregnancy outcomes and gynecological diseases. Microorganisms.

[64] Saadaoui M., Singh P., Al Khodor S. (2021). Oral microbiome and pregnancy: a bidirectional relationship. J. Reprod. Immunol..

