# Supplementary material for: Association between labor epidural analgesia and gut microbiota: A prospective cohort study
Source: Heliyon. 2024 Apr 21;10(9):e29883. doi: 10.1016/j.heliyon.2024.e29883 (PMC11064136; doi:10.1016/j.heliyon.2024.e29883)
Supplement: Multimedia component 4 [file mmc4.pdf]

目录

1. **Diet over the past week**......1
2. **36-item Pregnancy Stress Rating Scale (PSRS36)**......1
3. **Pregnancy Related Anxiety Questionnaire-Revised (PRAQ-R)**......3
4. **Numerical rating scale (NRS)**......3
5. **10-item Edinburgh Postnatal Depression Scale (EPDS)**......4
6. **Chinese version (中文版本)**......5
   1) 过去一周饮食情况......5
   2) 妊娠压力量表......5
   3) 妊娠相关焦虑量表（修订版）......6
   4) 数字疼痛评分......7
   5) 爱丁堡产后抑郁量表......7

1. Diet over the past week

Have you had any of the following eating habits in the past week？

Vegan diet	Fermented vegetables	Fried foods	Alcohol
□Yes □No	□Yes □No	□Yes □No	□Yes □No

2. 36-item Pregnancy Stress Rating Scale (PSRS36)

The following is a list of stressful situations you may encounter during pregnancy. Each item has five options, ranging from 0 to 4 (0 = none and 4 = very severe). Please tick " √ " in the corresponding box.

	**0**	**1**	**2**	**3**	**4**
1.Preparation of clothes and newborn supplies for baby					
2.Finding a qualified baby-sitter					
3.Choosing a place to ‘do-the-month’					
4.Naming my baby					
5.Acceptance of the child by significant others					
6.Doctor attitudes during labor and delivery					
7.Nurse attitudes during labor and delivery					
8.Deciding who will help take care of the baby					

9.Arranging for someone to handle housework during labor					
10.Husband's absence during labor					
11.Breast or bottle feed my baby					
12.Ability to breastfeed successfully					
13.Baby's gender					
14.Maternal behavior influencing the fetus					
15.Concern about status of fetal movement					
16.Sexual activity during pregnancy					
17.Baby's appearance					
18.Ability to raise my baby successfully					
19.Loss of free time after birth					
20.Safe delivery for my baby's sake					
21.Baby's health					
22.Safe delivery for my baby's sake					
23.Premature labor					
24.Abnormal or difficult birth					
25.Baby's birth weight					
26.Doctor may not arrive on time at delivery					
27.Unbearable labor pain					
28.Altered body shape during pregnancy					
29.Dark brown areas appearing on the skin					
30.Controlling weight during pregnancy					
31.Mobility difficulties due to altered body shape					
32.Returning to prenatal body shape and weight during postnatal period					
33.Sleep quality					
34.Adhering to traditional pregnancy mores					
35.Support from family members or husband					
36.Increased financial burden					

3. Pregnancy Related Anxiety Questionnaire-Revised (PRAQ-R)

If you have any of the following concerns during pregnancy, there are four options (1 = not at all and 4 = very much in items 1–5; 1 = never and 4 = all of the time in items 6–10). Please tick " √ " in the corresponding box.

	1	2	3	4
I am worried about the pain of contractions and the pain during delivery.				
I am anxious about the delivery because I have never experienced one before.				
I am worried about not being able to control myself during labor and fear that I will scream.				
I am afraid the baby will be mentally handicapped or will suffer from brain damage.				
I am afraid our baby will be stillborn, or will die during or immediately after delivery.				
I am afraid that our baby will suffer from a physical defect or worry that something will be physically wrong with the baby.				
I sometimes think that our child will be in poor health or will be prone to illnesses.				
I am worried about the fact that I shall not regain my figure after delivery.				
I am concerned about my unattractive appearance.				
I am worried about my enormous weight gain.				

4. Numerical rating scale (NRS)

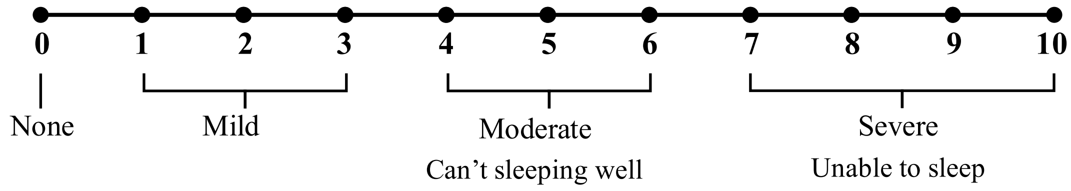

5. 10-item Edinburgh Postnatal Depression Scale (EPDS)

As you have recently had a baby, we would like to know how you are feeling. Please tick " √ " in the box which comes closest to how you have felt in the past days, not just how you feel today.

Situation	Feeling
I have been able to laugh and see the funny side of things	□A. As much as I always could □B. Not quite so much now<br>□C. Definitely not so much now □D. Not at all
I have looked forward with enjoyment to things	□A. As much as I ever did □B. Rather less than I used to<br>□C. Definitely less than I used to □D. Hardly at all
I have blamed myself unnecessarily when things went wrong	□A. Yes, most of the time □B. Yes, sometimes<br>□C. Not very often □D. No, never
I have been anxious or worried for no good reason	□A. No, not at all □B. Hardly ever<br>□C. Yes, sometimes □D. Yes, very often
I have felt scared or panicky for no very good reason	□A. Yes, quite a lot □B. Yes, sometimes<br>□C. No, not much □D. No, not at all
Things have been getting on top of me	□A. Yes, most of the time I havent been able to cope at all<br>□B. Yes, sometimes I havent been coping as well as usual<br>□C. No, most of the time I have coped quite well<br>□D. No, have been coping as well as ever
I have been so unhappy that I have had difficulty sleeping	□A. Yes, most of the time □B. Yes, sometimes<br>□C. Not very often □D. No, not at all
I have felt sad or miserable	□A. Yes, most of the time □B. Yes, quite often<br>□C. Not very often □D. No, not at all
I have been so unhappy that I have been crying	□A. Yes, most of the time □B. Yes, quite often<br>□C. Only occasionally □D. No, not at all
The thought of harming myself has occurred to me	□A. Yes, quite often □B. Sometimes<br>□C. Hardly ever □D. Never

6. Chinese version (中文版本)
1) 过去一周饮食情况

过去一周，您有如下饮食习惯吗？

纯素食	发酵/腌渍蔬菜	油炸食物	酒精
☐是 ☐否	☐是 ☐否	☐是 ☐否	☐是 ☐否

2) 妊娠压力量表

下面所列的是您在怀孕期间可能会遇到的会使您产生压力的情况，每个条目有 5 个选择项，从 0 分到 4 分代表压力从没有到很严重。请根据实际情况在对应的框内打“ √ ”

	0	1	2	3	4
1.准备婴儿的衣服及用品有困难					
2.找到一个满意的保姆有困难					
3.选定坐月子的地方有困难					
4.给孩子取名字有困难					
5.担心重要的他人不能接受孩子					
6.担心分娩时医生的态度					
7.担心分娩时护士的态度					
8.决定谁来帮助照顾孩子有困难					
9.在分娩期间不能安排好家务					
10.担心分娩时丈夫不在					
11.决定婴儿喂养方式有困难					
12.担心能否顺利哺乳					
13.担心孩子的性别与预期不符					
14.担心自己的孕期行为习惯影响胎儿					
15.担心胎动异常					
16.影响性生活					
17.担心孩子的外貌					
18.担心孩子的抚养问题					
19.担心生孩子后自己的自由时间变少					
20.担心孩子能否安全娩出					
21.担心孩子的健康情况					
22.担心自己分娩是否安全					
23.担心早产					

24.担心分娩可能出现不正常的情况或者转为剖宫产					
25.担心孩子的出生体重					
26.担心分娩时医生不能按时赶到					
27.担心分娩疼痛难以忍受					
28.担心体形改变					
29.担心脸上出现妊娠斑					
30.担心体重大幅增加					
31.担心不能控制笨拙的身体					
32.担心产后难以恢复产前的身材					
33.担心产后睡眠质量					
34.担心传统的孕期习俗对胎儿产生影响					
35.担心有孩子后会影响与丈夫或其他家庭成员感情					
36.担心不能给孩子提供良好的生活条件					

3) 妊娠相关焦虑量表（修订版）

妊娠期间，您是否有以下担心，每个条目有 4 个选择项，从 1 分到 4 分代表压力从没有到很严重。请根据实际情况在对应的框内打“ √ ”

	**1**	**2**	**3**	**4**
我担心宫缩的疼痛和分娩时的疼痛。				
我对分娩感到焦虑，因为我以前从未经历过。				
我担心分娩时无法控制自己，害怕我会尖叫。				
我担心生的孩子的智力有缺陷。				
我担心孩子怀孕期间或分娩期间死亡。				
我担心生的孩子身体上可能有缺陷，如畸形。				
我担心生的孩子身体不好或容易生病。				
我担心产后身材改变。				
怀孕期间，我担心容貌改变。				
怀孕期间，我担心体重大幅增加。				

4) 数字疼痛评分

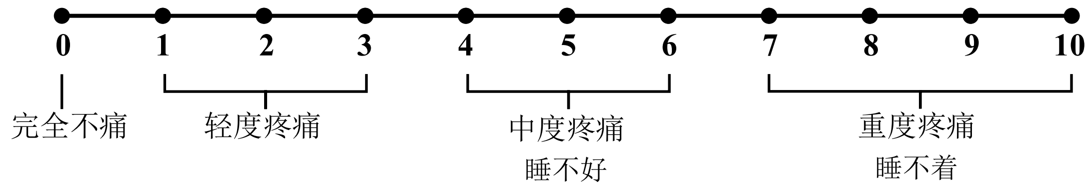

5) 爱丁堡产后抑郁量表

我们想知道你产后感觉如何。请在最接近你过去的感受的方框上打“√”

情景	感受
我看到有趣的事物能够开怀大笑。	□A. 跟以前一样 □B. 可能比以前少点<br>□C. 肯定比以前少 □D. 几乎没有
我怀着愉快的心情期待着一切	□A. 跟以前一样 □B. 可能比以前少点<br>□C. 肯定比以前少 □D. 几乎没有
当事情出错时，我无故责备自己	□A. 大多数时候 □B. 有时 □C. 不经常 □D. 从未
我一直无故焦虑或担心	□A. 从未 □B. 几乎不 □C. 有时 □D. 经常
我曾无故感到害怕或恐慌	□A. 多次 □B. 有时 □C. 不经常 □D. 从未
我感觉事情把我压垮了	□A. 是的，大多数时候我根本处理不来<br>□B. 是的，有时候我不像平时那样处理自如<br>□C. 不，大多数时候我处理得很好<br>□D. 不，我和以前一样能处理
我一直很不高兴以致失眠	□A. 大多数时候 □B. 有时 □C. 不经常 □D. 从未
我感到悲伤或痛苦	□A. 大多数时候 □B. 有时 □C. 不经常 □D. 从未
我一直很不高兴，经常哭泣	□A. 大多数时候 □B. 有时 □C. 偶尔 □D. 从未
我曾有过自残的念头	□A. 大多数时候 □B. 有时 □C. 几乎不 □D. 从未
