# Supplementary material for: Association between labor epidural analgesia and gut microbiota: A prospective cohort study
Source: Heliyon. 2024 Apr 21;10(9):e29883. doi: 10.1016/j.heliyon.2024.e29883 (PMC11064136; doi:10.1016/j.heliyon.2024.e29883)
Supplement: Multimedia component 5 [file mmc5.docx]

**Table S1.** Results of LEfSe from the phylum to species levels

| Taxonomy | LEfSe | | | |
| --- | --- | --- | --- | --- |
|  | **Group** | **LDA score** | ***P* value** | **adjusted *P* value** |
| *Maternal* |  |  |  |  |
| *p__Bacteroidota* | C | 4.03 | 0.012 | 0.014 |
| *c__Alphaproteobacteria* | C | 4.12 | 0.036 | 0.036 |
| *c__Bacteroidia* | C | 4.02 | 0.012 | 0.014 |
| *g__unidentified_Clostridia;s__gut_metagenome* | E | 4.23 | <0.001 | <0.001 |
| *g__unidentified_Clostridia;s__metagenome* | C | 4.46 | 0.012 | 0.014 |
| *s__Acinetobacter_pittii* | C | 4.49 | <0.001 | <0.001 |
| *s__Escherichia_coli* | E | 4.71 | <0.001 | 0.002 |
| *s__Pseudomonas_aeruginosa* | C | 4.16 | <0.001 | <0.001 |
| *s__Romboutsia_ilealis* | E | 4.09 | <0.001 | <0.001 |
| *s__Stenotrophomonas_maltophilia* | C | 4.02 | <0.001 | <0.001 |
| *Neonatal* |  |  |  |  |
| *c__Alphaproteobacteria* | C1 | 4.00 | 0.019 | 0.023 |
| *c__Bacilli* | C1 | 4.45 | 0.002 | 0.005 |
| *o__Lactobacillales* | C1 | 4.38 | <0.001 | <0.001 |
| *f__Lactobacillaceae* | C1 | 4.29 | <0.001 | <0.001 |
| *f__Muribaculaceae* | C1 | 4.44 | <0.001 | 0.001 |
| *f__Ruminococcaceae* | E1 | 4.33 | 0.038 | 0.038 |
| *g__Faecalibacterium* | E1 | 4.33 | 0.031 | 0.033 |
| *g__Lactobacillus* | C1 | 4.19 | <0.001 | <0.001 |
| *g__unidentified_Prevotellaceae* | E1 | 4.04 | 0.006 | 0.008 |
| *s__Bacteroides_fragilis* | C1 | 4.27 | 0.006 | 0.008 |
| *g__unidentified_Prevotellaceae;s__metagenome* | E1 | 4.28 | 0.001 | 0.004 |
| *s__Faecalibacterium_prausnitzii* | E1 | 4.43 | 0.011 | 0.014 |

*p*, phylum; *c*, class; *o*, order; *f*, family; *g*, genus; *s*, species; LDA, linear discriminant analysis

**Table S2.** Results of Metastats from the phylum to species levels in mothers

|  | Group | *P* value | adjusted *P* value |
| --- | --- | --- | --- |
| *p_Armatimonadota* | C | 0.004 | 0.040 |
| *p_Deferribacteres* | C | 0.003 | 0.040 |
| *p_**Hydrogenedentes* | C | <0.001 | 0.020 |
| *c_Campylobacterota* | C | 0.002 | 0.035 |
| *c_Chthonomonadetes* | C | <0.001 | 0.024 |
| *c_unidentified_ Acidobacteriota* | C | <0.001 | 0.024 |
| *c_unidentified_Deferribacteres* | C | 0.003 | 0.043 |
| *c_unidentified_ Firmicutes* | C | <0.001 | 0.024 |
| *o_Acholeplasmatales* | C | <0.001 | 0.012 |
| *o_Campylobacterales* | C | 0.002 | 0.021 |
| *o_Chthonomonadales* | C | <0.001 | <0.001 |
| *o_Clostridiales* | E | <0.001 | <0.001 |
| *o_Corynebacteriales* | C | <0.001 | <0.001 |
| *o_Deferribacterales* | C | 0.003 | 0.026 |
| *o_Flavobacteriales* | C | <0.001 | <0.001 |
| *o_Gastranaerophilales* | C | <0.001 | <0.001 |
| *o_Lactobacillales* | C | 0.003 | 0.026 |
| *o_Micrococcales* | C | <0.001 | 0.012 |
| *o_Rhizobiales* | C | 0.003 | 0.026 |
| *o_Rhodospirillales* | C | <0.001 | 0.012 |
| *o_Solibacterales* | C | <0.001 | 0.012 |
| *o_Sphingomonadales* | C | 0.002 | 0.021 |
| *o_Thermoanaerobacterales* | C | <0.001 | 0.012 |
| *o_unidentified_Acidobacteriota* | C | <0.001 | 0.012 |
| *f_Acetobacteraceae* | C | 0.002 | 0.020 |
| *f_Acholeplasmataceae* | C | <0.001 | 0.012 |
| *f_Aerococcaceae* | C | <0.001 | 0.012 |
| *f_Aeromonadaceae* | C | <0.001 | 0.012 |
| *f_Alcaligenaceae* | C | <0.001 | 0.012 |
| *f_Campylobacteraceae* | E | <0.001 | 0.012 |
| *f_Carnobacteriaceae* | C | <0.001 | 0.012 |
| *f_Christensenellaceae* | C | <0.001 | 0.012 |
| *f_Chthonomonadaceae* | C | <0.001 | 0.012 |
| *f_Clostridiaceae* | E | <0.001 | 0.012 |
| *f_Comamonadaceae* | C | 0.002 | 0.020 |
| *f_Deferribacteraceae* | C | 0.003 | 0.028 |
| *f_Flavobacteriaceae* | C | <0.001 | 0.012 |
| *f_Hafniaceae* | C | <0.001 | 0.012 |
| *f_Halomonadaceae* | C | <0.001 | 0.012 |
| *f_Helicobacteraceae* | C | <0.001 | 0.012 |
| *f_Lactobacillaceae* | C | 0.005 | 0.042 |
| *f_Micrococcaceae* | C | <0.001 | 0.012 |
| *f_Moraxellaceae* | C | <0.001 | 0.012 |
| *f_Nitrosomonadaceae* | C | 0.002 | 0.020 |
| *f_Nocardiaceae* | C | <0.001 | 0.012 |
| *f_Pasteurellaceae* | C | <0.001 | 0.012 |
| *f_Pectobacteriaceae* | C | 0.003 | 0.028 |
| *f_Peptostreptococcaceae* | E | 0.004 | 0.035 |
| *f_Propionibacteriaceae* | C | <0.001 | 0.012 |
| *f_Pseudoalteromonadaceae* | C | 0.004 | 0.035 |
| *f_Rhizobiaceae* | C | 0.002 | 0.020 |
| *f_Solibacteraceae* | C | <0.001 | 0.012 |
| *f_Sphingomonadaceae* | C | 0.002 | 0.020 |
| *f_unidentified_Acidobacteriota* | C | <0.001 | 0.012 |
| *f_unidentified_Rhodospirillales* | C | <0.001 | 0.012 |
| *f_unidentified_Thermoanaerobacterales* | C | <0.001 | 0.012 |
| *f_Vibrionaceae* | C | <0.001 | 0.012 |
| *f_Weeksellaceae* | C | <0.001 | 0.012 |
| *f_Xanthomonadaceae* | C | <0.001 | 0.012 |
| *g_Achromobacter* | C | <0.001 | 0.015 |
| *g_Acidaminococcus* | E | <0.001 | 0.015 |
| *g_Acinetobacter* | C | <0.001 | 0.015 |
| *g_Adhaeribacter* | C | 0.002 | 0.025 |
| *g_Aerococcus* | C | <0.001 | 0.015 |
| *g_Aeromonas* | C | <0.001 | 0.015 |
| *g_Alkalibacterium* | C | <0.001 | 0.015 |
| *g_Allobaculum* | E | 0.003 | 0.031 |
| *g_Alloprevotella* | C | <0.001 | 0.015 |
| *g_Anaerolinea* | C | 0.004 | 0.038 |
| *g_Anaeroplasma* | C | <0.001 | 0.015 |
| *g_Bosea* | C | 0.002 | 0.025 |
| *g_Brevibacillus* | C | <0.001 | 0.015 |
| *g_Brevundimonas* | C | <0.001 | 0.015 |
| *g_Brucella* | C | <0.001 | 0.015 |
| *g_Buchnera* | E | <0.001 | 0.015 |
| *g_Calorithrix* | C | <0.001 | 0.015 |
| *g_Campylobacter* | E | <0.001 | 0.015 |
| *g_Chthonomonas* | C | <0.001 | 0.015 |
| *g_Colidextribacter* | E | 0.003 | 0.031 |
| *g_Comamonas* | C | <0.001 | 0.015 |
| *g_Cutibacterium* | C | <0.001 | 0.015 |
| *g_Dubosiella* | E | <0.001 | 0.015 |
| *g_Elizabethkingia* | C | <0.001 | 0.015 |
| *g_Exiguobacterium* | C | 0.005 | 0.046 |
| *g_Floricoccus* | C | <0.001 | 0.015 |
| *g_Geobacillus* | C | 0.003 | 0.031 |
| *g_Glutamicibacter* | C | <0.001 | 0.015 |
| *g_Haemophilus* | C | 0.002 | 0.025 |
| *g_Halomonas* | C | <0.001 | 0.015 |
| *g_Helcococcus* | C | <0.001 | 0.015 |
| *g_Helicobacter* | C | <0.001 | 0.015 |
| *g_Herbaspirillum* | C | <0.001 | 0.015 |
| *g_Holdemanella* | C | <0.001 | 0.015 |
| *g_Intestinimonas* | E | <0.001 | 0.015 |
| *g_Kurthia* | C | 0.002 | 0.025 |
| *g_Lactococcus* | E | 0.005 | 0.046 |
| *g_Lautropia* | C | <0.001 | 0.015 |
| *g_Micrococcus* | C | 0.004 | 0.038 |
| *g_Microvirgula* | C | <0.001 | 0.015 |
| *g_Mobiluncus* | E | 0.003 | 0.031 |
| *g_Mucispirillum* | C | 0.003 | 0.031 |
| *g_Neisseria* | C | 0.004 | 0.038 |
| *g_Odoribacter* | C | <0.001 | 0.015 |
| *g_Olsenella* | E | 0.002 | 0.025 |
| *g_Paracoccus* | C | 0.003 | 0.031 |
| *g_Parvibacter* | E | 0.002 | 0.025 |
| *g_Pectobacterium* | C | 0.003 | 0.031 |
| *g_Pediococcus* | C | 0.002 | 0.025 |
| *g_Photobacterium* | C | <0.001 | 0.015 |
| *g_Pseudoalteromonas* | C | 0.004 | 0.038 |
| *g_Pseudoxanthomonas* | C | <0.001 | 0.015 |
| *g_Ramlibacter* | C | <0.001 | 0.015 |
| *g_Rhodococcus* | C | <0.001 | 0.015 |
| *g_Romboutsia* | E | 0.003 | 0.031 |
| *g_Roseomonas* | C | <0.001 | 0.015 |
| *g_Rothia* | C | <0.001 | 0.015 |
| *g_Segetibacter* | E | 0.003 | 0.031 |
| *g_Senegalimassilia* | C | <0.001 | 0.015 |
| *g_Sphingomonas* | C | 0.003 | 0.031 |
| *g_Stenotrophomonas* | C | <0.001 | 0.015 |
| *g_Thermoanaerobacterium* | C | <0.001 | 0.015 |
| *g_Turicibacter* | C | 0.003 | 0.031 |
| *g_unidentified_Acidobacteriota* | C | <0.001 | 0.015 |
| *g_unidentified_Beijerinckiaceae* | C | <0.001 | 0.015 |
| *g_unidentified_Flavobacteriaceae* | C | <0.001 | 0.015 |
| *g_unidentified_Micropepsaceae* | C | 0.002 | 0.025 |
| *g_unidentified_Rhodospirillales* | C | <0.001 | 0.015 |
| *g_unidentified_Ruminococcaceae* | C | 0.002 | 0.025 |
| *g_Vibrio* | C | <0.001 | 0.015 |
| *g_Weissella* | C | 0.004 | 0.038 |
| *s_Acinetobacter_bereziniae* | C | <0.001 | 0.013 |
| *s_Acinetobacter_pittii* | C | <0.001 | 0.013 |
| *s_Acinetobacter_sp_CIP_64_7* | C | <0.001 | 0.013 |
| *s_Actinomyces_oris* | C | <0.001 | 0.013 |
| *s_Adlercreutzia_caecicola* | E | 0.002 | 0.022 |
| *s_Aerococcus_urinaeequi* | C | <0.001 | 0.013 |
| *s_Agrobacterium_radiobacter* | C | <0.001 | 0.013 |
| *s_Alistipes_inops* | C | <0.001 | 0.013 |
| *s_Bacteroides_barnesiae* | C | 0.004 | 0.036 |
| *s_Bacteroides_clarus* | E | 0.003 | 0.029 |
| *s_Bifidobacterium_commune* | C | <0.001 | 0.013 |
| *s_Brevundimonas_diminuta* | C | <0.001 | 0.013 |
| *s_Brucella_melitensis* | C | <0.001 | 0.013 |
| *s_Christensenella_minuta* | E | 0.006 | 0.049 |
| *s_Chryseobacterium_indologenes* | C | 0.005 | 0.043 |
| *s_Clostridia_bacterium_UC5_1_1E11* | E | 0.002 | 0.022 |
| *s_Clostridiales_bacterium_CIEAF_020* | C | 0.002 | 0.022 |
| *s_Clostridiales_bacterium_enrichment_culture_clone_06_1235251_76* | C | <0.001 | 0.013 |
| *s_Clostridium_sp_Marseille_P3244* | C | <0.001 | 0.013 |
| *s_Comamonas_testosteroni* | C | <0.001 | 0.013 |
| *s_Corynebacterium_glucuronolyticum* | C | <0.001 | 0.013 |
| *s_Elizabethkingia_anophelis* | C | <0.001 | 0.013 |
| *s_Firmicutes_bacterium_M10_2* | E | 0.002 | 0.022 |
| *s_Floricoccus_penangensis* | C | <0.001 | 0.013 |
| *s_Geobacillus_stearothermophilus* | C | 0.003 | 0.029 |
| *s_Kurthia_sp_11kri321* | C | 0.002 | 0.022 |
| *s_Lactobacillus_agilis* | C | 0.006 | 0.049 |
| *s_Lactobacillus_delbrueckii* | C | <0.001 | 0.013 |
| *s_Lactobacillus_johnsonii* | C | 0.003 | 0.029 |
| *s_Lautropia_mirabilis* | C | <0.001 | 0.013 |
| *s_Massiliprevotella_massiliensis* | C | <0.001 | 0.013 |
| *s_Methylorubrum_extorquens* | C | <0.001 | 0.013 |
| *s_Micrococcus_luteus* | C | 0.004 | 0.036 |
| *s_Microvirgula_aerodenitrificans* | C | <0.001 | 0.013 |
| *s_Mucispirillum_sp_69* | C | 0.003 | 0.029 |
| *s_Pectobacterium_carotovorum_subsp_brasiliense* | C | 0.003 | 0.029 |
| *s_Photobacterium_damselae* | C | <0.001 | 0.013 |
| *s_Prevotella_corporis* | E | 0.004 | 0.036 |
| *s_Prevotella_melaninogenica* | C | 0.005 | 0.043 |
| *s_Pseudomonas_aeruginosa* | C | <0.001 | 0.013 |
| *s_Pseudomonas_oryzihabitans* | C | <0.001 | 0.013 |
| *s_Pseudomonas_parafulva* | C | <0.001 | 0.013 |
| *s_Rhodococcus_erythropolis* | C | <0.001 | 0.013 |
| *s_Romboutsia_ilealis* | E | 0.003 | 0.029 |
| *s_Roseomonas_gilardii* | C | <0.001 | 0.013 |
| *s_Ruminococcus_flavefaciens* | C | <0.001 | 0.013 |
| *s_Sphingomonas_paucimobilis* | C | <0.001 | 0.013 |
| *s_Stenotrophomonas_acidaminiphila* | C | <0.001 | 0.013 |
| *s_Stenotrophomonas_maltophilia* | C | <0.001 | 0.013 |
| *s_Stenotrophomonas_rhizophila* | C | <0.001 | 0.013 |
| *s_Stenotrophomonas_sp_G4* | C | <0.001 | 0.013 |
| *s_Vibrio_parahaemolyticus* | C | 0.006 | 0.049 |
| *s_Weissella_cibaria* | C | 0.005 | 0.043 |
| *g_Alloprevotella;s_gut_metagenome* | C | 0.004 | 0.036 |
| *g_Faecalibacterium;s_metagenome* | C | <0.001 | 0.013 |
| *g_Moryella;s_human_gut_metagenome* | E | 0.003 | 0.029 |
| *g_Ruminococcus;s_metagenome* | E | <0.001 | 0.013 |
| *g_Sutterella;s_gut_metagenome* | E | 0.002 | 0.022 |
| *g_unidentified_Acidobacteriota;s_metagenome* | C | <0.001 | 0.013 |
| *g_unidentified_Christensenellaceae;s_human_gut_metagenome* | C | 0.002 | 0.022 |
| *g_unidentified_Clostridia;s_metagenome* | C | <0.001 | 0.013 |
| *g_unidentified_Flavobacteriaceae;s_gut_metagenome* | C | <0.001 | 0.013 |
| *g_unidentified_Lachnospiraceae;s_mouse_gut_metagenome* | E | <0.001 | 0.013 |
| *g_unidentified_Micropepsaceae;s_metagenome* | C | 0.002 | 0.022 |
| *g_unidentified_Oscillospiraceae;s_human_gut_metagenome* | C | <0.001 | 0.013 |
| *g_unidentified_Rhodospirillales;s_gut_metagenome* | C | <0.001 | 0.013 |
| *g_unidentified_Ruminococcaceae;s_gut_metagenome* | C | <0.001 | 0.013 |

*p*, phylum; *c*, class; *o*, order; *f*, family; *g*, genus; *s*, species

**Table S3.** Results of Metastats from the phylum to species levels in neonates

|  | Group | *P* value | adjusted *P* value |
| --- | --- | --- | --- |
| *p_Deferribacteres* | C1 | 0.003 | 0.039 |
| *p_Kapabacteria* | C1 | <0.001 | 0.019 |
| *p_Spirochaetota* | E1 | <0.001 | 0.019 |
| *c_Bacilli* | C1 | 0.003 | 0.049 |
| *c_Sericytochromatia* | C1 | <0.001 | 0.032 |
| *c_Spirochaetia* | E1 | <0.001 | 0.032 |
| *c_unidentified_Deferribacteres* | C1 | 0.003 | 0.049 |
| *f_Lactobacillaceae* | C1 | <0.001 | 0.042 |
| *f_Leptotrichiaceae* | C1 | <0.001 | 0.042 |
| *f_Muribaculaceae* | C1 | <0.001 | 0.042 |
| *f_Orbaceae* | C1 | <0.001 | 0.042 |
| *f_Peptostreptococcaceae* | E1 | <0.001 | 0.042 |
| *f_**Spirochaetaceae* | E1 | <0.001 | 0.042 |
| *f_unidentified_Bacteria* | C1 | <0.001 | 0.042 |
| *g_Algoriphagus* | C1 | <0.001 | 0.037 |
| *g_Azospirillum* | C1 | <0.001 | 0.037 |
| *g_Blastomonas* | C1 | <0.001 | 0.037 |
| *g_Citrifermentans* | C1 | <0.001 | 0.037 |
| *g_Gilliamella* | C1 | <0.001 | 0.037 |
| *g_Helcococcus* | E1 | <0.001 | 0.037 |
| *g_Lactobacillus* | C1 | <0.001 | 0.037 |
| *g_Lentimicrobium* | C1 | <0.001 | 0.037 |
| *g_Ligilactobacillus* | C1 | <0.001 | 0.037 |
| *g_Muribaculum* | C1 | <0.001 | 0.037 |
| *g_Mycoplasma* | E1 | <0.001 | 0.037 |
| *g_Nannocystis* | C1 | <0.001 | 0.037 |
| *g_Romboutsia* | E1 | <0.001 | 0.037 |
| *g_Snodgrassella* | C1 | <0.001 | 0.037 |
| *g_Sphingobium* | C1 | <0.001 | 0.037 |
| *g_Sporacetigenium* | C1 | <0.001 | 0.037 |
| *g_Treponema* | E1 | <0.001 | 0.037 |
| *g_unidentified_Bacteria* | C1 | <0.001 | 0.037 |
| *g_Algoriphagus* | C1 | <0.001 | 0.037 |
| *g_Azospirillum* | C1 | <0.001 | 0.037 |
| *g_Blastomonas* | C1 | <0.001 | 0.037 |
| *g_Citrifermentans* | C1 | <0.001 | 0.037 |
| *g_Gilliamella* | C1 | <0.001 | 0.037 |
| *g_Helcococcus* | E1 | <0.001 | 0.037 |
| *g_Lactobacillus* | C1 | <0.001 | 0.037 |
| *g_Lentimicrobium* | C1 | <0.001 | 0.037 |
| *g_Ligilactobacillus* | C1 | <0.001 | 0.037 |
| *g_Muribaculum* | C1 | <0.001 | 0.037 |
| *g_Mycoplasma* | E1 | <0.001 | 0.037 |
| *g_Nannocystis* | C1 | <0.001 | 0.037 |
| *g_Romboutsia* | E1 | <0.001 | 0.037 |
| *g_Snodgrassella* | C1 | <0.001 | 0.037 |
| *g_Sphingobium* | C1 | <0.001 | 0.037 |
| *g_Sporacetigenium* | C1 | <0.001 | 0.037 |
| *g_Treponema* | E1 | <0.001 | 0.037 |
| *g_unidentified_Bacteria* | C1 | <0.001 | 0.037 |
| *s_Adlercreutzia_caecicola* | E1 | 0.002 | 0.035 |
| *s_Anaerotignum_lactatifermentans* | E1 | <0.001 | 0.023 |
| *s_Bilophila_wadsworthia* | E1 | <0.001 | 0.023 |
| *s_Campylobacter_jejuni* | E1 | <0.001 | 0.023 |
| *s_Caulobacter_sp* | C1 | 0.002 | 0.035 |
| *s_Clostridiales_bacterium_CCNA10* | E1 | <0.001 | 0.023 |
| *s_Clostridiales_bacterium_CIEAF_020* | C1 | <0.001 | 0.023 |
| *s_Clostridium_sp_Culture_41* | C1 | 0.002 | 0.035 |
| *s_Dialister_pneumosintes* | E1 | <0.001 | 0.023 |
| *s_Floricoccus_penangensis* | C1 | 0.002 | 0.035 |
| *s_Gilliamella_apicola* | C1 | <0.001 | 0.023 |
| *s_Kofleria_flava* | C1 | <0.001 | 0.023 |
| *s_Lactobacillus_delbrueckii* | C1 | 0.002 | 0.035 |
| *s_Lactobacillus_johnsonii* | C1 | <0.001 | 0.023 |
| *s_Lactobacillus_reuteri* | C1 | 0.002 | 0.035 |
| *s_Megasphaera_stantonii* | E1 | <0.001 | 0.023 |
| *s_Moryella_sp_KHD1* | C1 | 0.003 | 0.046 |
| *s_Mucispirillum_sp_69* | C1 | 0.003 | 0.046 |
| *s_Muribaculum_intestinale* | C1 | <0.001 | 0.023 |
| *s_Nannocystis_pusilla* | C1 | <0.001 | 0.023 |
| *s_Photobacterium_damselae* | C1 | 0.003 | 0.046 |
| *s_Prevotella_sp_AG_487_50_53* | E1 | <0.001 | 0.023 |
| *s_Romboutsia_ilealis* | E1 | <0.001 | 0.023 |
| *s_Ruminococcus_gnavus* | C1 | <0.001 | 0.023 |
| *s_Sphingomonas_paucimobilis* | C1 | <0.001 | 0.023 |
| *s_Sporacetigenium_mesophilum* | C1 | <0.001 | 0.023 |
| *s_Stenotrophomonas_rhizophila* | C1 | <0.001 | 0.023 |
| *s_TM7_phylum_sp_oral_clone_CW040* | C1 | <0.001 | 0.023 |
| *g_Desulfovibrio;s_metagenome* | E1 | 0.002 | 0.035 |
| *g_Lachnoclostridium;s_human_gut_metagenome* | E1 | <0.001 | 0.023 |
| *g_Prevotella;s_gut_metagenome* | E1 | <0.001 | 0.023 |
| *g_unidentified_Anaerolineae;s_metagenome* | C1 | <0.001 | 0.023 |
| *g_unidentified_Bacilli;s_gut_metagenome* | C1 | 0.003 | 0.046 |
| *g_unidentified_Muribaculaceae;s_mouse_gut_metagenome* | C1 | <0.001 | 0.023 |
| *g_unidentified_Prevotellaceae;s_human_gut_metagenome* | C1 | 0.003 | 0.046 |
| *g_unidentified_Ruminococcaceae;s_human_gut_metagenome* | E1 | <0.001 | 0.023 |

*p*, phylum; *c*, class; *o*, order; *f*, family; *g*, genus; *s*, species
